# Evaluating the effectiveness of Hong Kong’s border restriction policy in reducing COVID-19 infections

**DOI:** 10.1186/s12889-022-13234-5

**Published:** 2022-04-21

**Authors:** Pengyu ZHU, Xinying TAN

**Affiliations:** 1 Associate Professor Division of Public Policy, Hong Kong University of Science and Technology, Hong Kong SAR, China; 2 PhD Student in Public Policy, Division of Public Policy, Hong Kong University of Science and Technology, Hong Kong SAR, China

**Keywords:** Travel restriction, Human mobility, COVID-19 pandemic, Synthetic control method, Hong Kong

## Abstract

This study evaluates the effectiveness of Hong Kong’s strict border restrictions with mainland China in curbing the transmission of COVID-19. Combining big data from Baidu Population Migration with traditional meteorological data and census data for over 200 Chinese cities, we utilize an advanced quantitative approach, namely synthetic control modeling, to produce a counterfactual “synthetic Hong Kong” without a strict border restriction policy. We then simulate infection trends under the hypothetical scenarios and compare them to actual infection numbers. Our counterfactual synthetic control model demonstrates a lower number of COVID-19 infections than the actual scenario, where strict border restrictions with mainland China were implemented from February 8 to March 6, 2020. Moreover, the second synthetic control model, which assumes a border reopen on 7 May 2020 demonstrates nonpositive effects of extending the border restriction policy on preventing and controlling infections. We conclude that the border restriction policy and its further extension may not be useful in containing the spread of COVID-19 when the virus is already circulating in the local community. Given the substantial economic and social costs, and as precautionary measures against COVID-19 becomes the new normal, countries can consider reopening borders with neighbors who have COVID-19 under control. Governments also need to closely monitor the changing epidemic situations in other countries in order to make prompt and sensible amendments to their border restriction policies.

**Supplementary Information:**

The online version contains supplementary material available at 10.1186/s12889-022-13234-5.

## Introduction

Cross-border population movement in much of the world has come to a standstill as jurisdictions impose restrictions on international travel to contain the abrupt spread of the new coronavirus disease 2019, or COVID-19. As of March 31, 2020, over 90 percent of the global population, amount to around 7.1 billion people, live in countries that restrict entry of incoming passengers who are neither residents nor citizens [1]. To limit cross-border transmission of the virus, Hong Kong instituted mandatory quarantine for travelers from mainland China, the earliest epicenter, soon after the epidemic hit its communities, but this border control measure provoked disputes as social and commercial interactions between the two sides were disrupted. At the early stages of the outbreak, countries and territories around the world imposed different forms of travel restrictions with regard to mainland China, including border closure which is defined as a partial or total closure of a land border with China [2]. Restriction of population movement across the border connecting Hong Kong and the mainland is unprecedented. These restrictions were implemented through the imposition of a 14-day mandatory quarantine for mainland travelers on February 8, 2020. The quarantine order led to a de facto border closure with the mainland as it effectively denied entry to the city for mainland visitors who generally hold a visa / entry permit with a 7-day permitted period of stay. The policy was extended several times beyond its initial expiration date on May 7, and it is still in force as of the time of writing this paper. In this study, we refer to this quarantine measure as a strict border restriction policy with mainland China. Hong Kong is not the only jurisdiction to use mandatory quarantines as a means to restrict travel; more than a dozen states in the U.S. have also imposed quarantine orders for inter-state travelers to avoid transmitting the virus [3].

Considering the high contagion and great uncertainties of COVID-19, importation of infections is a major risk factor of the epidemic [4]. However, limiting cross-border travel not only leads to reduced interactions across all economic sectors but also interrupts the delivery of medical aid and technical support that are vital to stem the spread of virus [5–7]. Therefore, this study intends to investigate whether Hong Kong’s strict border restriction policy for mainland travelers and its further extensions were effective and necessary in containing the spread of COVID-19 in Hong Kong. Specifically, we examine whether the border restriction policy reduced the epidemic size, as measured by the number of daily new and cumulative infections. Our evaluation applies an advanced synthetic control method in comparative study to estimate the real policy effects, bridging qualitative application and quantitative inference in policy analysis. Using human mobility big data provided by one of China’s largest internet company, Baidu, and combined with city basic statistics, we construct a counterfactual model as a combination of Chinese cities with no strict inter-city travel restriction and simulate the epidemic trajectory in this synthetic control counterfactual.

## Background

**Fig. 1:**
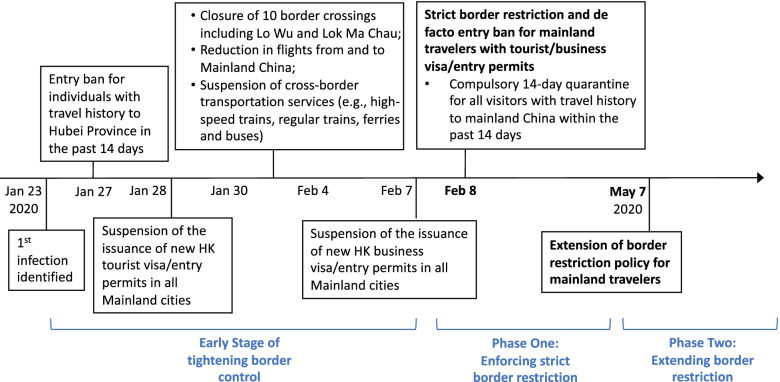
Timeline of the incremental implementation of HK’s border restriction policy with mainland China

The COVID-19 outbreak has challenged health security and created public panic in Hong Kong. On January 23, 2020, the city confirmed its first COVID-19 infection, an imported case from Wuhan, China [8, 9]. Subsequently, three more cases were classified as local cases on February 4 after epidemiological investigations [10], which indicated the occurrence of local community transmission. By the end of March 2020, Hong Kong had reported 682 confirmed infections with four deaths.^1^ As the situation evolved rapidly, the Hong Kong (HK) government implemented a series of non-pharmaceutical interventions and policy measures, including border restrictions, to stem the virus transmission.^2^ Throughout the epidemic development, Hong Kong’s border restriction and control measures with mainland China have been enhanced incrementally. Here, we divided the policy implementation process into three stages. A historical record of the escalation of relevant policy measures is shown in Fig. 1.

During the early stage of local outbreak, the HK government tightened its border controls with some hotspot cities in mainland China. The government announced on January 27 that all visitors from Hubei Province or travelers who have travel history to Hubei in the past 14 days would be barred from entry [11]. Along with the restriction of entry for this group of people, the administration also requested a halt in the issuance of new tourist visa/entry permit to Hong Kong in all mainland cities starting from January 28 [12]. The issuance of new business visa/entry permits to Hong Kong was also suspended soon after. Starting from January 30, the government further tightened its border controls with measures including closing several land border crossings, reducing the number of flights to and from mainland China, and suspending all cross-border bus, train and ferry services [11]. These actions mark the early stage of border control (between January 23 and February 7).

Efforts for border control culminated in a policy of mandatory 14-day quarantine for all visitors with travel history to mainland China within the past 14 days, effective February 8 [11, 13]. Beginning February 8, Hong Kong entered Phase One of the implementation of border restrictions with a strict entry ban for most mainland travelers. Under this policy, all mainland visitors with visas valid less than 14 days are effectively denied entry to Hong Kong. This category includes all Chinese tourists and business travelers. The strict border restrictions that took effect on February 8 have slowed travel between Hong Kong and the mainland to a trickle. According to data from the Immigration Department, after implementation of the strict entry restriction and mandatory home-based quarantine measures, the number of people entering Hong Kong via border control points and the airport dropped by 73% in one and an half months, from 121,828 people as recorded on February 4, to 32,216 on March 18 [14]. In contrast, most cities in mainland China did not impose strict inter-city travel bans, except Wuhan and a few other cities in Hubei Province. Additionally, mainland cities have generally adopted less disruptive nonpharmaceutical interventions (NPIs), such as centralized quarantine, access control for gated communities, social distancing, and contact tracing (e.g., using Health Code) to limit the risks of transmission of COVID-19. Each of these policy choices comes with different social and economic implications relative to the alternatives. As border restriction policies bring with them substantial costs stemming from major reductions in cross-border economic activities, the *first* objective of this research is to evaluate the effectiveness of strict border restrictions with the mainland China in terms of limiting COVID-19 infections in Hong Kong.

As planned, the administrative order of 14-day quarantine mainland travelers after entering Hong Kong would expire on May 7. However, the HK government chose to extend its strict border restriction policy beyond the initial expiration date,^3^ at a time when most places in mainland China had the epidemic under control. Thus, we designate the period starting from May 7 as Phase Two of Hong Kong’s border restriction policy. As expected, this policy extension continued to suppress cross-border economic activities. Our *second* objective is therefore to examine whether this extension was effective and necessary to further contain the epidemic, given that the number of confirmed infections in nearly all mainland cities has remained very low, with no inter-city travel restrictions or quarantine requirements for domestic travelers within the mainland.

The findings of this research will help the Hong Kong government to revisit its border restriction policies, and to improve its governance of public health and risk management in handling other infectious diseases in the future. More importantly, containment strategies come with significant social and economic costs. As many countries that have implemented border restrictions and quarantine orders are considering reopening their economies, this study gives a useful framework for anti-pandemic policy evaluation and decision making.

## Literature review

### Impacts of travel restrictions on COVID-19 control

The use of non-pharmaceutical interventions (NPIs) was proven to help prevent COVID-19 infections but the effectiveness of different NPIs varied [15]. Currently, the impact of cross-border travel restrictions are not well understood due to the challenges of defining and encoding them [16, 17]. Moreover, the evidence from the limited number of studies is mixed. Some studies suggest that such measures can delay and constrain virus spread [18], whereas others argue that they have negligible effects on overall case numbers [19]. Early studies paid attention to the large-scale and stringent travel restrictions implemented in mainland China, such as the cordon sanitaire imposed in Wuhan on 23 January 2020 [20–22]. Since the start of the outbreak, cities of Hubei Province including Wuhan have implemented inter-city travel restrictions or lockdown policies, with relative success with regard to containing the spread of the epidemic [23–25]. Clinical research suggests that Wuhan’s measures to control mobility reduced the median daily reproduction number (${R}_{t}$) of the disease after one week [26]. The cordon sanitaire introduced in Wuhan limited the geographical transmission of COVID-19 across China, and international travel restrictions also slowed the spread in other places in the world [22, 27]. However, others also found that inter-city travel restrictions alone had no substantial effect on the progression of epidemic in some major cities in China (e.g., Beijing, Shenzhen), but had some positive effects in small cities [28]. China’s efforts in restricting intercity mobility amidst the COVID-19 outbreak was particularly effective in containing the virus within hotspot areas, but when the virus spread beyond these boundaries, its efficacy decreased [20]. Data from cities in China and the U.S. indicate the association between population density and the COVID-19 infection rate, which supports the importance of travel restrictions in preventing virus spread [29]. Considering the externality of an individual’s travel, Oum & Wang suggests that travel restrictions should be stricter and haver higher violation penalties in places with high population density [30]. Evidence from Latin America also supports the need for COVID-19 control to restrict air travel, especially in air transportation hubs [31]. Apart from inter-city travel restrictions, limiting intra-city mobility or city lockdowns also contributed to overall containment of the epidemic [22].

Travel restrictions may be implemented together with other prevention and control measures. Lai et al. estimated that a combination of NPIs (e.g., early detection and isolation of infected cases, social distancing and contact reduction) prevented more COVID-19 infections than did travel restrictions [15]. A review of quarantine effects concluded that, combining quarantine with travel restrictions and other prevention and control measures shows a larger effect than any individual measures in terms of reducing infectious disease case numbers and mortality, including for COVID-19, SARS, and MERS [32]. Previous research provides evidence for the effectiveness of NPIs. Markel et al. studied several NPIs (i.e., cancellation of public gatherings, school closure, and isolation/quarantine) used by cities in the U.S. under the hit of influenza pandemic in 1918–1919 [33]. Their results demonstrated a strong association between the application of NPIs and the mitigation of the pandemic in terms of reducing mortality.

As the COIVD-19 pandemic evolves, it is important to evaluate and update these containment policies implemented in Hong Kong. In addition, since Hong Kong is not the origin or epicenter of this pandemic, this research may provide a useful reference for other jurisdictions which face similar challenges from both virus spread and current hotspot areas to their local communities.

### Influence of human mobility on the transmission of epidemic diseases

Human mobility is one of the most important drivers of the spread of epidemic diseases [34–36]. When confronted with life-threatening infectious diseases (eg: SARS, H1NA, MERS), governments often impose controls that restrict or constrain population mobility [37, 38]. The goal of these measures is to protect non-infected areas from the epidemic by suspending travel from and to the areas with an active outbreak. Research has generally indicated that restrictions on human mobility can slow down the propagation of epidemics [39]. Indeed, this is the fundamental reason for governments to implement various containment policies to reduce mobility in fighting pandemics. Recent research has investigated the relationship between human mobility and the transmission of COVID-19. Human mobility data gives a precise record of how infections were distributed spatially in the country when the outbreak had just begun in China[20]. In the early stage of the outbreak, several studies found a significant association between air and rail travel and the number of COVID-19 infections [40–42]. The lockdown of Wuhan and other cities in Hubei Province was proven to be effective in containing the spread of the epidemic across China [24, 43, 44]. COVID-19 is transmissible in community settings, and local clusters of infections in Singapore are expected to be linked to travel flow from China before the Wuhan lockdown [45]. A global study using a spatial–temporal network model with network dynamics showed there could be correlations between international human mobility and the epidemic situations in hotspot areas[46]. More recently, a novel study proposed an asymmetric spatial weight matrix based on population flow in China during the pandemic [47]. The authors then applied this matrix in spatial econometric modelling and found that population inflow from Wuhan were strongly correlated to COVID-19 infections. According to these studies, we believe that human mobility plays a vital role in the transmission of COVID-19.

## Method

We combine population migration big data from Baidu with daily meteorological data and yearly city statistics and use an advanced quantitative approach, namely, the Synthetic Control Method (SCM), to reproduce a synthetic control unit from an optimal weighted linear combination of over 200 cities, as a counterfactual. We are able to verify that the pre-intervention characteristics of the synthetic controls are similar to those of Hong Kong. With the constructed synthetic controls, we estimate the epidemiological trends of COVID-19 transmission for two counterfactual scenarios: 1) the strict border restriction policy with the mainland was not imposed on February 8; 2) there was a border reopening and lift off mandatory quarantine measures on May 7 (i.e., there was no extension of the strict border restriction policy after its original expiration date). We use these synthetic controls to simulate counterfactual COVID-19 infection trajectories. Specifically, daily new infections and cumulative infections will be estimated in such synthetic controls, predicted by the aggregate COVID-19 data of the untreated donor cities. By comparing actual confirmed case numbers with those in our counterfactual models where Hong Kong had hypothetically imposed no border restrictions, we evaluate the effectiveness of Hong Kong’s border restriction policy and its extension. In our previous study, we also applied the SCM approach to evaluate the effects of compulsory home quarantine for inbound travelers [48].

We have set the control periods for the Phase One and Phase Two analyses to be the 14 days prior to their respective intervention dates (i.e., Jan 25-Feb 7 in Phase One; and April 23-May 6 in Phase Two). To capture the policy effects, for Phase One, we constrain the post-treatment period to the 28 days (4 weeks) after the start of border restriction policy on Feb 08, i.e., Feb 8-Mar 6. For the Phase Two analysis, the post-treatment period for the policy extension is between May 07 and May 31.

## Data

We incorporate a number of predictor variables into our SCM models, including epidemiological variables (i.e., the past-14-day moving average of daily reported cumulative confirmed infections, and the past-14-day moving average of cumulative infections per 10,000 people), inter-city population movements (i.e., daily volume of inter-city travel, derived from Baidu Migration Big Data), city-level demographics and socio-economics, and natural meteorological parameters. Historical realization of new/cumulative infections and the aforementioned predictor variables, which are relevant in determining the level of infections, allow us to simulate and predict outbreak trajectories in the counterfactual settings. The descriptive statistics for our sample are provided in the Appendix. Specifically, epidemiological variables and population movements are measured daily; city-level demographic and socio-economic variables are year-round statistics; and natural meteorological parameters are measured with daily average values for each city. For most of these variables, the data follow a normal distribution. Note that epidemiological variables are highly skewed to the right because a few hotspot cities had server situations and reported extreme case numbers for some days.

## Baidu Migration Big Data

As the frequency of inter-regional travel hypothetically affects the rate of transmission of a pandemic, we utilize the data from Baidu Population Migration to capture trip frequency from and to each city. Baidu Population Migration Big Data is a database developed by Baidu, Inc. in 2014. This database applies location-based service (LBS) technology to record and visualize the movement trajectories of mobile internet users throughout the country. Baidu LBS technology is used in many cellphone apps, including Baidu Map and thousands of third-party applications. In China, 80% of all mobile phones have one or more applications with Baidu LBS installed [49]. With positioning information automatically fed to Baidu servers, real-time location records are collected and analyzed on the cloud computing platform to generate intercity and intracity real-time travel information, which is then formulated into the Baidu Migration Indices, representing the level of in-migration index and out-migration in each city. To convert the two indexes into the actual volume of person-movements in and out of each city, we use the daily number of people flowing into and out of Hong Kong provided by the Hong Kong Immigration Department to calibrate and calculate how many person-movements correspond to one unit of the in-migration index and out-migration index. After linking the two sets of daily mobility data of Hong Kong, we run an OLS regression on the number of actual population movement within the period and estimate that one index unit is equal to around 71,121 person-movements. We use this estimate to calculate the real volume of daily population inflows and outflows of each city throughout the sampling period.

Most people in mainland China use mobile internet actively for various purposes, including communication, entertainment, and navigation. Baidu Migration Big Data captures a vast proportion of individual movements across cities within China, making it the most comprehensive and accurate representation of intercity human movements in China. We have derived all data since January 1, 2020, for 363 Chinese cities including Hong Kong and Macao.

## COVID-19 epidemiological data

The COVID-19 Epidemic Spatial–Temporal Dataset provides a multi-scale dataset tracking the COVID-19 global epidemic from December 31, 2019, onwards. The project extracts epidemiological data for China from reports provided by China’s national, provincial and municipal Health Committees. We retrieved the epidemiological data from this dataset and obtained the daily reported numbers of new infections and cumulative infections for each prefecture-level city in the mainland, Macao, and Hong Kong from January 11 to May 31, 2020. In order to capture the changing dynamics of epidemiological trends, we calculated the past-14-day moving average of cumulative infections and the past-14-day moving average of cumulative infections per 10,000 people. The latter takes into account the influence of population size. We utilize the two main epidemiological variables as predictors in our SCM model.

### City-level demographic and socio-economic variables

We consider the influence of city demographics, including year-round average population, number of households, population density, in our model estimation. It is arguable that patterns of sociodemographic dependence could exist and affect the transmission of COVID-19. For example, a study found significant strong correlations between COVID-19 cases and population numbers in 60.0% of the regions in Italy [50]. A study in the U.S. suggested that contact rates were higher in counties with greater population density and thus, contributed to higher transmission rate, measured by the basic reproductive number (R0), of COVID-19 [51]. Also, it is suggested by a recent study that characteristics of business services and associated human activities in cities can reflect the level of COVID-19 infection risks [52]. The city basic parameters are drawn from the city Statistical Yearbook provided by each city’s respective Census and Statistics Departments. We also incorporate several predictor variables to control for socioeconomic factors in our empirical model, including level of economic development (represented by total GDP, per capital GDP) and public health capacity (represented by the number of hospitals, per-capital hospital beds, and number of medics per 10,000 people).

### City-level natural meteorological parameters

Various natural meteorological parameters, such as mean temperature, wind speed, relative humidity, and air quality index (AQI), are included as predictors. After verifying the normality of these parameters, we adopt the daily average values when measuring these factors for each city. There is evolving research which substantiates that these factors could have influence on the transmissibility of contagious diseases. COVID-19 is classified as a seasonal low-temperature infection that transmits through airborne pathways, and is negatively affected by temperature and absolute humidity [53]. It was suggested that higher temperature and relative humidity are associated with reductions in the effective reproductive number (R value) and suppress the transmission of COVID-19 in both China and the U.S [54]. A study in Korea found a significant but positive association between daily temperature and the number of daily COVID-19 cases[55]. It was also found that slower outdoor wind speed is associated with increased risk of COVID-19 transmission [56]. Moreover, numerous studies have found that ambient air pollutants (e.g., SO_2_, NO_2_, CO), particulate matter (PM), PM2.5 and PM10 can affect COVID-19 epidemiology in various geographical regions [57], although some thresholds might exist [50, 55, 57, 58].

Meteorological data for mainland China were derived from the Meteorological Data Service Centre of China, which provides hourly records of temperature, relative humidity, and wind speed at each meteorological observatory station. Each variable was averaged over 24 h for each day of calculation to come up with the daily numbers. Empirical Bayesian Kriging (EBK) was used as the interpolation method to compute the values of the three meteorological elements in this study. EBK automates the difficult aspects of building a valid kriging model, taking the uncertainty of semivariogram estimation into account to optimize the simulations and predictions [59]. It uses an intrinsic random function as the kriging model, which can inherently correct for trends in the data. The application of EBK normally has some assumptions (e.g., stationarity, normality, absence of outliers) [60]. In practice, we realized this method following the instructions on ArcGIS^4^ for choosing semivariogram that provides the best fit to the empirical semivariances. For Hong Kong and Macao, we gathered daily meteorological data directly from the Hong Kong Observatory and the Macao Meteorological and Geophysical Bureau.

An assessment of city air quality can be made by using the Air Quality Index (AQI), which examines the levels of six atmospheric pollutants (e.g., SO2, PM2.5, PM10) across all detecting stations within every city. Data on AQI for each Chinese city are derived from Harvard Dataverse. Each record includes information on the daily average, maximum, minimum, and standard deviation value of AQI. In this study, we use the daily average AQI to represent the air quality conditions of each city. Meanwhile, daily AQI in the two special administration areas are obtained through the World Air Quality Index (WAQI), which provides AQI data for every air quality monitoring station. The values of daily AQI in Hong Kong (or Macao) are calculated by averaging the daily data of all air quality monitoring stations in the region.

## Empirical model

The setting of the SCM model is as shown below:

Suppose that data are obtained for $J$+1 cities: $j=\mathrm{1,2},\dots ,J+1$, and the first city $j=1$ is the treated city, that is, the city implementing a strict border restriction policy (i.e., Hong Kong). The other cities $j=2,\dots ,J+1$ are a cluster of cities without strict inter-city travel restrictions, which is a set of potential comparisons (i.e., the “donor pool”). Then, we suppose that our data cover phases $T$ and the phases ahead of policy implementation are denoted as ${T}_{0}$. In our analysis of Phase I, ${T}_{0}=$ February 8, when Hong Kong started to implement a strict border restriction policy with the mainland. In our Phase II analysis, ${T}_{0}=$ May 7, the date when Hong Kong extended border restrictions for mainland travelers.

Further, our outcomes of interest will be observed as ${Y}_{jt}$ for each city $j$ at time $t$. In this study, two major variables of interest, the daily reported number of cumulative COVID-19 infections, and the number of daily new COVID-19 infections, are to be estimated, respectively. For every city $j$, a series of $k$ predictors ${X}_{1j},\dots ,{X}_{kj}$ are determined for the two outcomes, which include the past-14-day moving average of cumulative infections, the past-14-day moving average of infections per 10,000 people, daily population inflow and outflow (only applicable to Phase One analysis), and city-level parameters that would not be affected by the border restriction policy (see Table 2 and 4 for a full list of predictor variables). According to Abadie [61], the predictors’ values for cities $j=1,\dots ,J+1$ are comprised by a $k\times 1$ vectors ${X}_{1},\dots ,{X}_{J+1}$, and the predictors’ values for the $J$ cities that have not been intervened are gathered by a $k\times J$ matrix, ${X}_{0}=\left[{X}_{2}\dots {X}_{J+1}\right]$. Then, we define ${Y}_{jt}^{N}$ as the potential response of COVID-19 infection numbers without strict border restriction for every city $j$ and time $t$. For Hong Kong ($j=1$) and a period after the treatment $t>{T}_{0}$, we describe ${Y}_{jt}^{I}$ as the observed outcomes. Then, the treatment effect of the border restriction policy in Hong Kong in period $t>{T}_{0}$ is given as:

1 $${{\uptau }_{1t}=Y}_{1t}^{I}-{Y}_{1t}^{N}$$

For the treated city Hong Kong, we can have ${Y}_{1t}={Y}_{1t}^{I}$. In this sense, the problem of estimating the counterfactual outcome ${Y}_{1t}^{N}$ is just equivalent to the challenge of estimating ${\tau }_{1t}$.

A synthetic control is referred to the weighted average of potential donor cities. It can be present by a $J\times 1$ vector of weights, $W = \left( {w_{2} , \ldots ,w_{J + 1} } \right)^{\prime }$. Abadie et al. [62, 63] proposed to select the synthetic control ${W}^{*}={\left({w}_{2}^{*}, \dots , {w}_{j+1}^{*}\right)}^{^{\prime}}$ so that it best approximates the treated city’s predictor and outcome data during the pre-intervention period. The optimal weights ${W}$ minimize

2 $$\Vert {X}_{1}-{X}_{0}W\Vert ={\left(\sum_{h=1}^{k}{\upsilon }_{h}{\left({X}_{h1}-{w}_{2}{X}_{h2}-\dots -{w}_{J+1}{X}_{hJ+1}\right)}^{2}\right)}^\frac{1}{2}$$

where the value of ${w}_{2},\dots ,{w}_{J+1}$ should be larger than zero and add up to one. Subsequently, the estimator of the treatment effect ${\tau }_{1t}$ for $j=1$ (i.e., Hong Kong) in period $t={T}_{0}+1,\dots ,T$ takes the following form:

3 $${\widehat{\uptau }}_{1t}={Y}_{1t}-\sum_{j=2}^{J+1}{w}_{j}^{*}{Y}_{jt}$$

The choice of $V=\left({\upsilon }_{1},\dots ,{\upsilon }_{k}\right)$ in Eq. (2) reproduce the value of the predictors ${X}_{11},\dots ,{X}_{k1}$, and simulate a synthetic control $W\left(V\right)={\left({w}_{2}\left(V\right),\dots ,{w}_{J+1}\left(V\right)\right)}^{^{\prime}}$ that minimizes the mean squared prediction error (MSPE) with respect to ${Y}_{1t}^{N}$:

4 $${\sum_{t\in {\mathcal{T}}_{0}}\left({Y}_{1t}-{\mathrm{w}}_{2}\left(V\right){Y}_{2t}-\dots -{\mathrm{w}}_{\mathrm{J}+1}\left(V\right){Y}_{\begin{array}{c}J\\ +1t\end{array}}\right)}^{2}$$

for $${\mathcal{T}}_{0}\subseteq \left\{\mathrm{1,2},\dots ,{T}_{0}\right\}$$.

For the period before policy intervention $t=\mathrm{1,2},\dots ,{T}_{0}$, the value of ${Y}_{1t}^{N}$ is identifiable. We then use the observed data to estimate the power of prediction on ${Y}_{1t}^{N}$ of the set of predictors. To achieve this, we use the *synth* command in Stata 15.0. The pre-intervention periods are divided evenly into a period for training and a period for validation. The weights of the synthetic control are calculated based on the data on all predictors in the training period. Then a value ${V}$ is obtained to minimize the MSPE and ${W}^{*}=W\left({V}^{*}\right)$ is computed by ${V}$ and all predictors’ data for the validation period.

## Empirical results

### The effectiveness of the strict border restriction policy for travelers from the mainland

In the baseline model in Panel A, the donor pool incorporates 282 cities, excluding Wuhan and other cities in Hubei Province where cordon sanitaire had been set up to restrict inter-city travel. As the timing and transmission speed of the outbreak were very different in different cities, we use a 14-day control period (i.e., between Jan 25 and Feb 7, 2020) to capture the variance in COVID-19 infection patterns across different cities. The sample weights obtained for the synthetic control unit are presented in Table 1. As demonstrated in Table 2, the simulated values of the predictors in the synthetic control unit are similar to the real values.

**Table 1:** Weights of donor cities for the synthetic control in Panel A and Panel B

Phase One: Implementation of strict border restriction policy for mainland travelers							
**Panel A (Fig. ****2****): Outcome: cumulative infections**		**Panel A (Fig. ****3****): Outcome: new infections**		**Panel B (Fig. ****4****): Outcome: cumulative infections**		**Panel B (Fig. ****5****): Outcome: new infections**	
**Donor city**	**Weight**	**Donor city**	**Weight**	**Donor city**	**Weight**	**Donor city**	**Weight**
Macau	0.619	Macau	0.292	Changzhou	0.544	Macau	0.289
Zhoushan	0.188	Qinzhou	0.278	Macau	0.434	Changzhou	0.204
Shuangyashan	0.152	Jiangmen	0.245	Dalian	0.015	Jiangmen	0.206
Shanghai	0.041	Zhoushan	0.082	Chongqing	0.007	Zhoushan	0.145
		Shanghai	0.052			Qinzhou	0.101
		Rikaze	0.037			Putian	0.033
		Yingkou	0.013			Chongqing	0.023
*Note*: The donor pool of Panel A excludes cities in Hubei Province with cordon sanitaire set up to restrict inter-city travel. The donor pool of Panel B further eliminates cities with different level of inter-city travel restriction measures. Sample weights are chosen to minimize the RMSPE of 14 days prior to the start of border restriction policies

**Table 2:** Model goodness of fit: balance of predictor variables between Hong Kong and the synthetic control unit during the pre-treatment period (Panel A and Panel B)

Phase One: Implementation of strict border restriction policy for mainland travelers					
		**Panel A**		**Panel B**	
**Outcome**		**Cumulative infections (Fig. ****2****)**	**new infections (Fig. ****3****)**	**cumulative infections (Fig. ****4****)**	**new infections (Fig. ****5****)**
**Predictor**	**Hong Kong**	**Synthetic HK**	**Synthetic HK**	**Synthetic HK**	**Synthetic HK**
# of Cumulative Infections (past-14-day moving average)	5.31	5.09	4.44	5.06	5.05
# of Infections per 10,000 people (past-14-day moving average)	0.0071	0.0442	0.0218	0.0315	0.0269
# of Population Inflow (preintervention average)	18,047	23,851	36,643	41,348	32,541
# of Population Outflow (preintervention, average)	14,390	17,004	26,790	31,327	28,179
Region GDP (million yuan)	2,398,046	383,279	339,391	510,875	328,213
GDP per capita (yuan)	321,842	369,638	211,467	323,292	231,524
# of people per sq.km)	6,737	13,677	6,834	9,996	6,824
# of Hospital Beds per 10,000 persons	54.27	47.19	46.74	45.96	50.99
# of Medics per 10,000 persons	19.66	30.33	29.90	33.99	33.63
Temperature (℃)	16.24	8.57	12.24	9.49	11.40
Relative Humidity (%)	72.50	75.40	75.40	77.84	77.81
Wind Speed (m/s)	8.80	3.64	3.15	2.97	3.12
Air Quality Index (AQI)	59.21	64.15	57.23	65.19	54.33
**RMSPE**		**1.0727**	**0.9194**	**1.1004**	**0.9969**

**Fig. 2:**
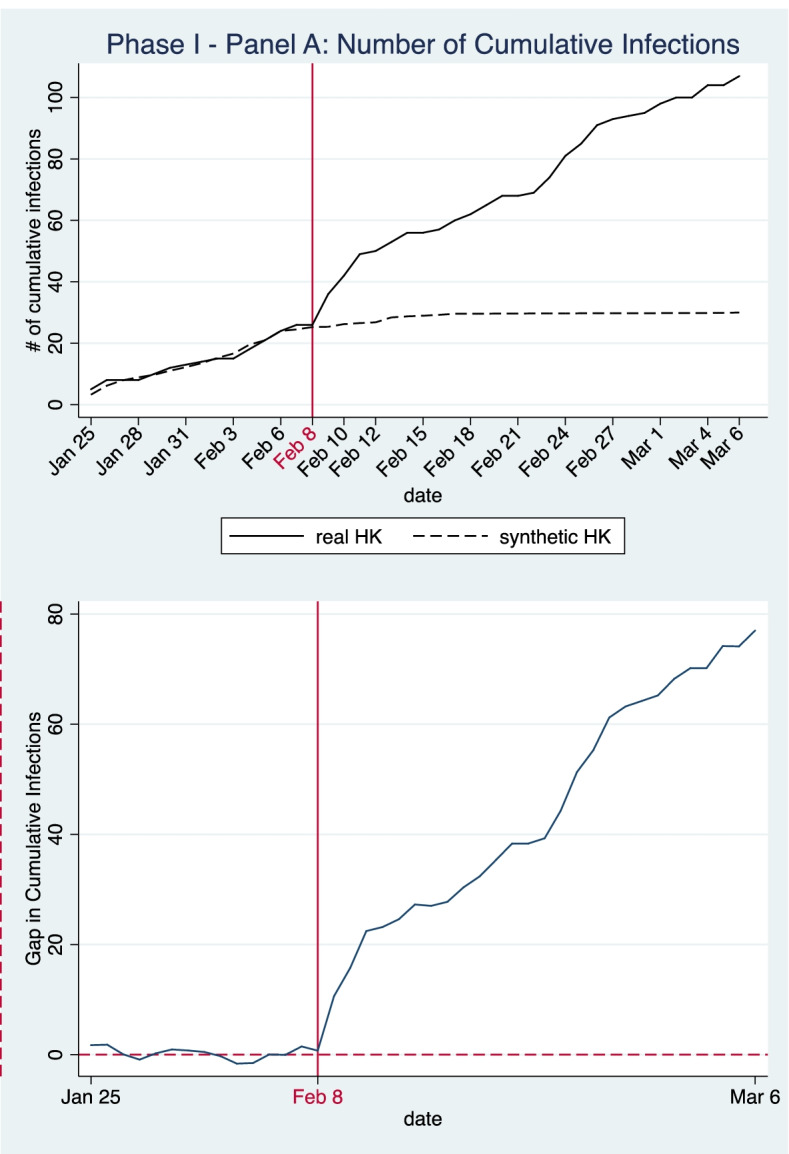
Panel A—Number of Cumulative Infections in Phase One

**Fig. 3:**
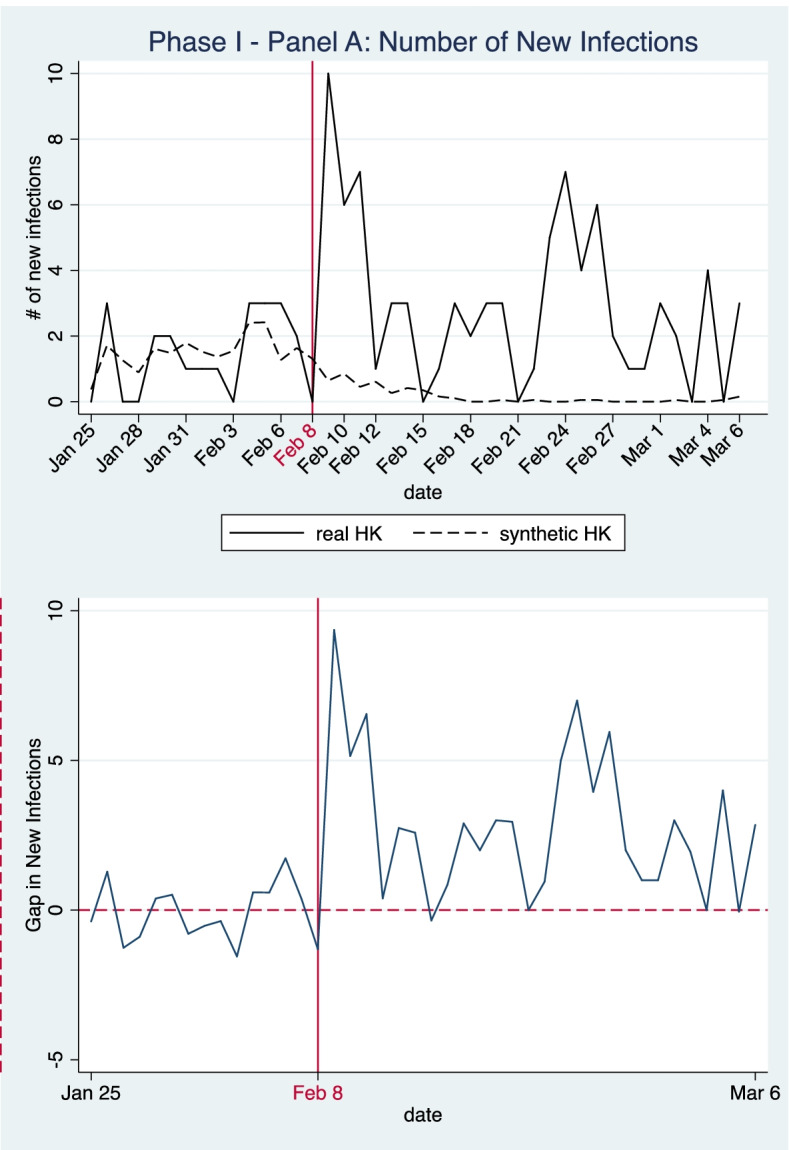
Panel A—Number of New Infections in Phase One

Panel A (Figs. 2 and 3) presents the baseline modeling results for the introduction of strict border restrictions with the mainland on February 8. Visual comparison of the development of cumulative/new confirmed infections shows that there is a good fit between the simulated trends in the synthetic control and the real trajectories during the pre-intervention period (i.e., before February 8). The two figures clearly show that during the policy intervention period, for both cumulative infections and daily new infections, the actual infection numbers in Hong Kong were generally higher than the counterfactual estimates in our simulation of a Hong Kong without border restrictions.

In Fig. 2, the difference in cumulative infections between the real HK and the synthetic Hong Kong after February 8 can be interpreted as the treatment effect of the border restriction policy. In this case, the treatment effect is positive and the gap between actual cumulative case numbers after the February 8 policy intervention and the corresponding counterfactual estimates gradually widens. During the first week of intervention, cumulative case numbers increase in both scenarios, but at a lower speed in the synthetic control. After mid-February, cumulative case numbers in the synthetic control stay at a moderate level, with just above 29 confirmed cases, while real epidemiological trends in post-intervention Hong Kong reflect an exacerbation of the outbreak. From February 8 to March 6, the actual cumulative case numbers increased from 26 to 107 with an average daily growth rate of 5.38%, while in the synthetic control, case numbers increased much more slowly, from 25.254 to 29.996 with an average daily growth rate of 0.64%.^5^ The actual daily growth rate of infections was thus 4.74 percentage points higher than the infection rate predicted in our simulation of a counterfactual Hong Kong without border restrictions. This result indicates that the strict border restriction policy implemented on February 8 did not effectively limit the infection number of COVID-19 in the city.

Figure 3 further compares daily new confirmed infections in the real HK and the synthetic control unit. The synthetic trend indicates that had Hong Kong not imposed border restrictions with the mainland on February 8 but instead implemented other extensive and stringent NPIs as in mainland cities, the number of daily new infections would have gradually diminished over the first 10 days, then drop close to 0 and stay at 0 from February 19 onwards. However, in reality, the number of new infections fluctuated widely and even reached as high as 10 infections in a single day. During the intervention period, the daily numbers of new confirmed infections actually observed in Hong Kong were in general higher than those estimated in our synthetic control unit. There are only three days (February 8, 15 and March 5) when the actual new case numbers were lower than the corresponding counterfactual estimates, but the net reduction was no more than 2 cases for the three days. Therefore, we conclude that the strict border restriction policy implemented on February 8 was not effective for controlling COVID-19 as compared with other extensive and stringent NPIs implemented in mainland cities.

We speculate two main reasons leading to the failure of this policy. First, its implementation was somewhat late as virus transmission in local communities had already been observed at the time of its implementation. When imported cases were reported, the virus had already crossed the border, and substantial risks associated with the local transmission chains. In addition to border restrictions, it was also very important to curb the local community transmission by intra-city control measures such as social distancing, contact tracing, erecting cordon in zones of substantial transmission. In the meantime, mainland cities have been focusing on blocking cross-community transmission by enforcing various types of prevention and control measures including body temperature checks at store entrances, access control for gated communities, social distancing, contact tracing, and so on. But these NPIs were not adopted or strictly enforced in Hong Kong during this time period.^6^ At a time when local transmission had already started, these NPIs would have been more effective than simply shutting down the border with the mainland. Secondly, Hong Kong did not implement a full-scale entry restriction to all overseas travelers in this early stage of policy intervention. Travelers from other countries or regions that were deemed “low risk” at that time might have contributed to the increase of infection cases in Hong Kong, whereas all international travelers were avoiding non-essential travel to mainland China by this time. Indeed, this speculation is supported by the fact that Hong Kong later observed a substantial increase in imported cases starting on March 6.

As some cities in our initial donor pool utilized various control measures to restrict the movement of people, including complete or partial lockdowns and the establishment of checkpoints and quarantine zones, incorporating those cities into our model might create confounding effects that bias our results. Therefore, we also checked the robustness of our baseline results by changing the donor pool. After reviewing the inter-city travel restrictions implemented in mainland cities, as documented by Fang et al. [21], we eliminated cities with different levels of inter-city travel restrictions during the study period from the donor pool. The resulting donor pool contains 210 prefecture-level cities. We then re-ran the SCM model using the same outcome variables and predictors. The results are shown in Panel B.

**Fig. 4:**
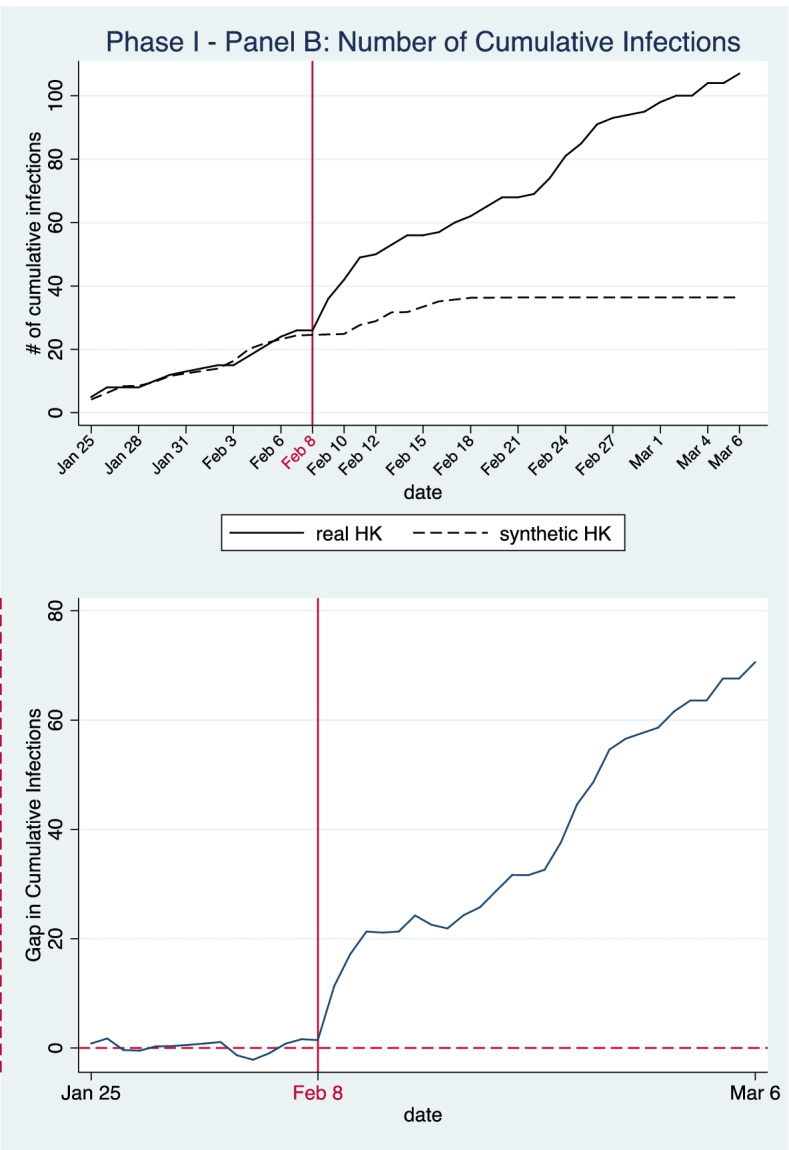
Panel B—Number of Cumulative Infections in Phase One

As indicated in Fig. 4, after adjustment of the donor pool, the actual cumulative case numbers remain higher than our counterfactual estimates during the intervention period. In our adjusted synthetic control, cumulative case numbers grew from 24.566 to 36.401 during the intervention period, an average daily infection growth rate of 1.47%; infection numbers stopped increasing after February 24. The actual daily growth rate of infections was 5.38%, much higher than the simulated counterfactual trend. Furthermore, cumulative case numbers by March 6 are higher in Panel B (36.401 cases) than in Panel A (29.996 cases). This is because the donor pool used in Panel B eliminated cities which had implemented control measures to limit inter-city travel and lower the risks of COVID-19 transmission. The results suggest that various types of inter-city travel restriction measures adopted in some mainland cities also had some impact when used together with other stringent NPIs.

**Fig. 5:**
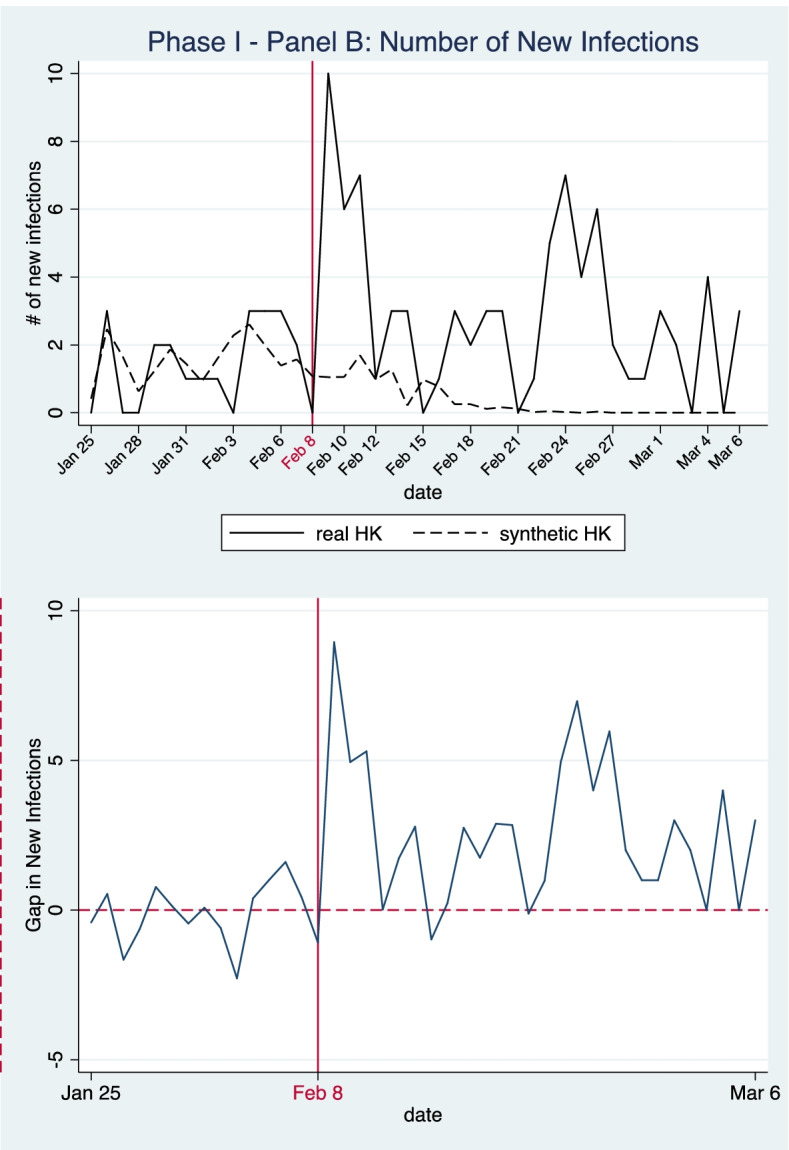
Panel B—Number of New Infections in Phase One

With respect to the patterns of daily new confirmed infections, the results in Fig. 5 are similar to those in Fig. 3 of Panel A. The actual numbers of daily new confirmed infections are in general higher than the corresponding estimates in our synthetic control unit. Actual daily new case numbers were only lower than the counterfactual estimates on February 8, 15 and 21, and the net difference for both days is just 2.172 cases. Apart from the three days, the strict border restriction policy failed to lower the number of daily new infections in Hong Kong, as compared with the counterfactual estimates. These results from this robustness check corroborate that the strict border restriction policy implemented on February 8 in Hong Kong was not as effective in containing the transmission of COVID-19 as other extensive NPIs adopted in mainland China.

## The effectiveness of the extension of the strict border restriction policy for mainland travelers

In Panel C, we simulate cumulative and daily new case numbers for a counterfactual policy scenario in which Hong Kong reopens its border with the mainland on 7 May 2020. The synthetic control is constructed with cities where no inter-city travel restrictions were in place after May 7. By this time, all cities in mainland China had lifted restrictions on inter-city travel and the donor pool in Panel C contains 282 cities. Note that we exclude the two migration variables (i.e., population in-flow and out-flow) as predictors in this estimation. During the investigation period from 23 April to 31 May 2020, the volume of population movement to and from Hong Kong was unusually low due to a series of travel alerts and restrictions, thus controlling for population migration in the SCM model would rule out many appropriate donors and confound the simulation and evaluation results. The weights of donor cities are illustrated in Table 3. As demonstrated in Table 4, the epidemic variables, as well as the socio-economic and natural meteorological characteristics of our synthetic controls are similar to the actual situations in Hong Kong.

**Table 3:** Weights of donor cities for synthetic control unit in Panel C

Phase Two: Extension of border restriction policies and compulsory home quarantine measure for mainland travellers			
**Panel C (Fig. ****6****): Outcome: cumulative infections**		**Panel C (Fig. ****7****): Outcome: new infections**	
**Donor city**	**Weight**	**Donor city**	**Weight**
Macau	0.378	Guangzhou	0.415
Guangzhou	0.31	Shanghai	0.316
Shanghai	0.241	Macau	0.255
Turfan	0.057	Wuhan	0.012
Wuhan	0.014	Huanggang	0.001

**Table 4:** Model goodness of fit: balance of predictor variables between Hong Kong and the synthetic control unit during the pre-treatment period (Panel C)

Phase Two: Extension of border restriction policies with mainland			
**Outcomes**		**Cumulative infections (Fig. ****6****)**	**new infections (Fig. ****7****)**
**Predictors**	**Real HK**	**Synthetic HK**	**Synthetic HK**
# of Cumulative Infections (14-day moving average)	1028.14	1029.40	1026.21
# of Infections per 10,000 people (14-day moving average)	1.3799	1.3456	1.3143
Region GDP (million yuan)	2,398,046	1,653,744	2,091,070
GDP per capita (yuan)	321,842	290,743	248,028
# of people per sq.km)	6,737	9,141	6,775
# of Hospital Beds per 10,000 persons	54.27	63.90	74.40
# of Medics per 10,000 persons	19.66	42.80	48.17
Temperature (℃)	24.68	22.47	22.33
Relative Humidity (%)	77.29	75.05	77.03
Wind Speed (m/s)	4.98	2.49	2.44
Air Quality Index (AQI)	62.71	56.56	52.08
**RMSPE**		**0.4458**	**0.6267**

**Fig. 6:**
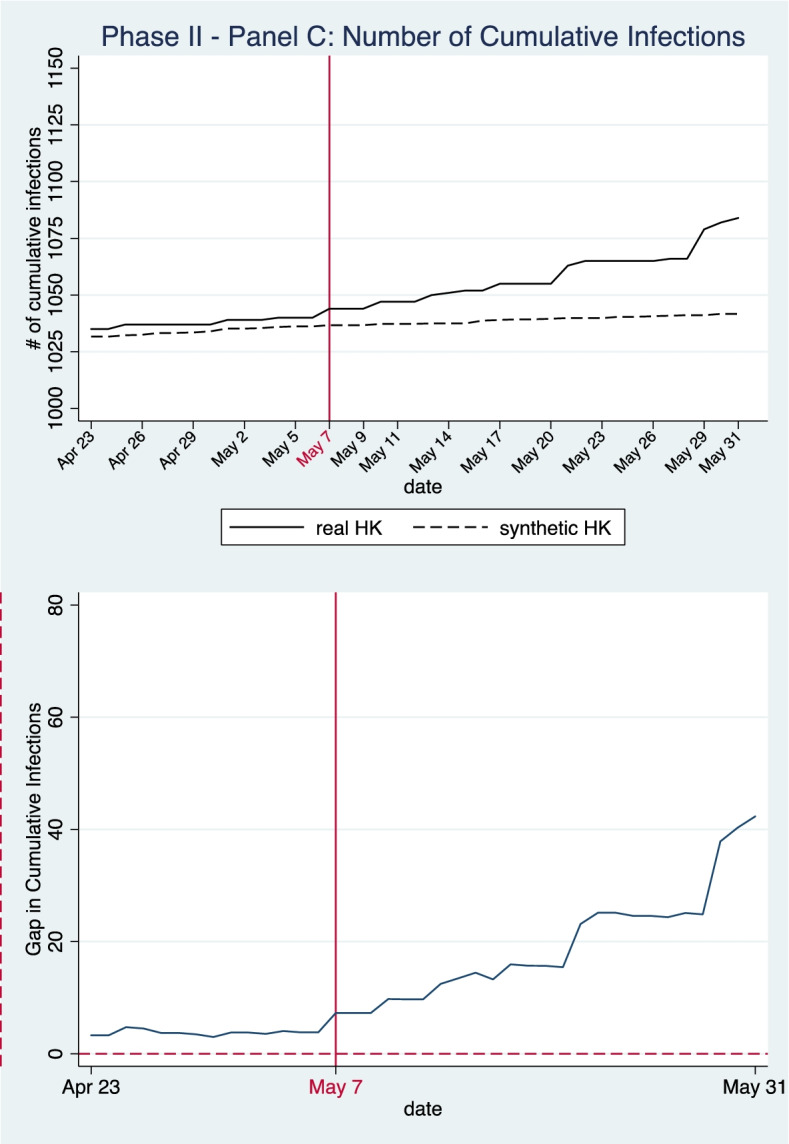
Panel C—Number of Cumulative Infections in Phase Two

Figure 6 shows that, as of May 7, cumulative infection numbers in the real HK were slightly higher but still very close to the trajectory of our synthetic control. This indicates the fitness of the simulation model. After May 7, Hong Kong maintained its strict border restriction policy for mainland travelers. The synthetic control in this test simulates infection trends in the counterfactual situation of a border reopening with the mainland on May 7. From the upper graph in Fig. 5, we can find there is parallel trend between HK and the synthetic control. However, the gap between the actual cumulative infection numbers and the simulated numbers widen gradually after May 7. To be more specific, cumulative infection numbers increased much faster in the real HK than in the synthetic control. The total number of cases in Hong Kong under continued border restrictions increased by 40 cases, or 3.83%, from May 7 to the end of May, whereas cumulative case numbers were only projected to increase by around 5 cases, or 0.48%, in our synthetic model of a border reopening on May 7. By the end of May, the number of infections in Hong Kong had reached 1084, whereas our synthetic model of Hong Kong given a lift of border restriction policy on May 7 shows only 1041.683 total infections by the same time period. This result suggests that the effectiveness of the extension of the strict border restriction policy for mainland travelers beyond May 7 was not very apparent.

**Fig. 7:**
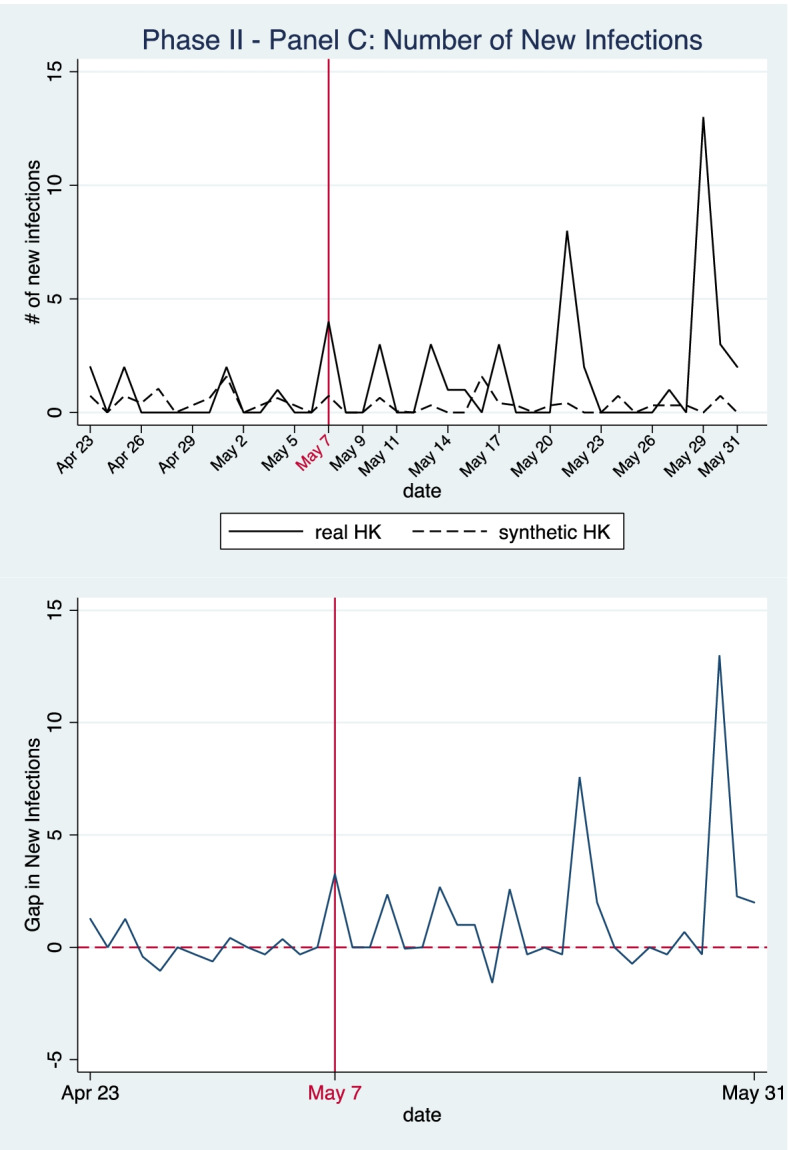
Panel C—Number of New Infections in Phase Two

Figure 7 shows our results for the daily number of new confirmed COVID-19 infections. We can see that the numbers of new infections under continued strict border restrictions fluctuate higher and lower than the simulated numbers in the synthetic control. The simulated trends suggest that had the strict border restriction policy been lifted on May 7, the outbreak would, in general, remain at a low level with no more than 1 new infections confirmed on any single day except the day of May 16 when the synthetic model projects a daily new infection number of 1.58. Despite the fact that HK achieved zero new infections for several days after the policy extension, the number of new infections reaches as high as 8 on May 21 and 13 on May 29, while numbers in the synthetic control remain close to zero. The comparison between the two scenarios reveals that the extension of the strict border restriction policy for mainland travelers from May 7 to June 7 failed to lower the number of daily new COVID-19 infections in Hong Kong. As the mainland has had the COVID-19 outbreak under control since early May, the decision to maintain strict border restrictions with the mainland was unnecessary and overcautious.

## Discussion

This study evaluates the effectiveness of Hong Kong’s border restriction policy with mainland China that introduced amidst the COVID-19 outbreak. Using daily epidemiological data, it compared Hong Kong’s border closure situation with the hypothetical control scenario simulated using mainland cities where inter-city travel was not restricted completely. Our results indicate that Hong Kong’s border restrictions with mainland China from February 08, 2020 may not have been very useful in containing the number of COVID-19 infections, when compared to the counterfactual scenario with no strict travel restrictions. The actual average daily growth rate of infections was 3.91–4.74 percentage points higher than the infection rates projected in our counterfactual models, despite the substantial drop in cross-border population movement as a result of the strict border restriction policy. This finding suggests that the effects of border restrictions or inter-city travel restrictions on COVID-19 control may not be significant when there is already an outbreak in the local communities. This is in contrast to recent medical research that argues border restrictions between Hong Kong and mainland China are effective in reducing daily travel volume, cumulative COVID-19 cases and mortality [64]. The authors used a metapopulation Susceptible-Exposed-Infectious-Recovered (SEIR) model with inspected migration to simulate and predict the epidemiological characteristics in Hong Kong after border closure. However, their estimation used retrospective data up to February 08, 2020, and did not take into account the real epidemiological trajectory after the implementation of border closure. Our study investigated the trends in confirmed case numbers during the pre- and post-treatment periods, and also considered the extension of the border restriction policy using actual data in April–May. Some studies in other regions of the world have similar findings to ours and suggest border restrictions might have minimal effect on COVID-19 transmission. For example, an interrupted time series study of nine African countries found an increase in the incidence rate of COVID-19 after border closure [65].

Our finding indicates that Hong Kong’s border restrictions with mainland China cannot help to control the risks of local community transmission. As there is evidence that community transmission occurred before February 8, we believe the delayed implementation of border restrictions with the mainland—after the local transmission had already begun—was one key problem reducing its effectiveness. Border restriction policies would have been more effective if implemented prior to transmission happening in local communities; otherwise, other less disruptive non-pharmaceutical interventions (NPIs) adopted internally within the city, such as body temperature checks at store entrance, access control for gated communities, social distancing, contact tracing, and quarantine of potential cases, may have been more effective than external containment measures such as border restrictions or entry bans. This result is in line with a recent study which found the effect of border closure between Switzerland and Italy could have been improved if implement two weeks earlier [66]. Such a finding is also supported by the results of a study [67] that assessed the effectiveness of more than 6,000 government interventions against COVID-19 with four computational techniques merging statistical, inference and artificial intelligence tools. Their results suggested the necessity of using a combination of various NPIs to contain the virus spread, and those less disruptive but costly NPIs could be as effective as the more invasive, drastic approaches including border closure and city lockdown. They also highlighted that the effectiveness of NPIs depends on the local context such as timing of their adoption. From the perspective of international travel restrictions, it is also suggested that border closure for air travel does not result in a considerable decline in the number of infection cases, but simpler preventative methods (e.g., preliminary PCR testing, mask use and contact tracing) can reduce the risk of COVID-19 importation from overseas [68, 69]. As border closures or restrictions often come with significant social and economic costs, policymakers should be cautious when implementing these policies. Of course, our conclusion is contingent on whether or not neighboring cities have implemented rigorous pharmaceutical treatments for the infected as well as various NPIs to actively curb local transmission. Had the neighboring cities or countries been negligent in disease control, travel restrictions and entry bans would have still been critical.

Despite the epidemic coming under control in most mainland cities, the HK government still decided to extend its strict border restriction with the mainland four times (i.e., extensions to June 7, July 7, August 7, and September 7 2020…). However, our second counterfactual estimation indicates nonpositive effects of such policy extensions and even suggests a lower number of COVID-19 infections if border restrictions had been lifted on their initial expiration data. Behavioral scientists have long contended that people tend to underreact before the outbreak of a shock (e.g., a contagious disease) and then overreact when the shock actually occurs. It is important for the government to review the current border restriction policy and make evidence-based decisions. When imposing border restrictions, government departments responsible for epidemic control should closely monitor the changing situations in the targeted places and other hotspot areas with observed or hidden high infection rate, in order to make prompt and sensible amendments toward the policy. Given that epidemic situation in the mainland was relatively stable, the HK government was overcautious in extending the border restriction policy, which also severely disrupted cross-border economic and social interactions. Similarly, other countries may also consider re-opening borders with neighbors who have COVID-19 under control.

In summary, our research provides evidence that the strict border restriction policy for travelers from the mainland may not have helped to curb the COVID-19 outbreak in Hong Kong at a time when transmission in local communities had already started and when nearly all mainland cities had already adopted rigorous pharmaceutical and non-pharmaceutical interventions. Moreover, we specifically look at the effectiveness of the extensions of the strict border restriction policy for mainland travelers. As precautionary measures against COVID-19 becomes the new normal, and given the hefty costs of the reduction in cross-border economic activities, there seems to be no justifiable reason for Hong Kong to continue its border restrictions with the mainland. These results may be useful to policymakers in Hong Kong and in other places around the world as they considering or exercising inter-city travel restrictions to limit the risks of COVID-19 transmission while seeking to control costs and gradually reopen their economies. Furthermore, the presented model is also suitable for analyzing the epidemiological characteristics of emerging variants of COVID-19 and other novel infectious diseases.

## Limitation of the study

We acknowledge that this study bears some limitations. First, it is unrealistic that the models adopted can include all confounding factors that may affect COVID-19 spread. As suggested in those references we reviewed, previous epidemiological conditions at different localities (e.g., population vulnerability due to other local specific comorbidities or compromised immune systems) may affect the new virus transmission, but they are not directly controlled in our simulation. Also, our models are unable to capture changes in social behavior (e.g., public panic) within local communities during the time, which could impose some uncertainties on epidemiological trajectory. Second, the robustness of our results may be subjected to the measurement of some variables. This study uses mean values to measure natural meteorological factors such as AQI, but COVID-19 transmission could be more influenced when these factors exceed certain threshold values (e.g., PM10 and PM2.5) as suggested by some research [57, 58]. Finally, statistical and mathematical approaches may not directly provide definitive causal evidence. When applying the results of this research, policymakers in different cities or regions should pay attention to their specific contexts and consider a set of available (or feasible) policy options jointly, rather than looking at each option separately. Nevertheless, this paper still provides essential evidence to the literature, especially when combined with other empirical studies.

## Supplementary Information

**Additional file 1:** Descriptive statistics

## Data Availability

The datasets used and/or analysed during the current study are available from the corresponding author on reasonable request.

## Footnotes

1 For details about the latest situation of cases of COVID-19 in Hong Kong (as of 31, March, 2020), see https://www.chp.gov.hk/files/pdf/local_situation_covid19_en_20200331_222412.pdf

2 The Legislative Council Panel on Health Services provided updates on the Government’s response and measures adopted to prevent and control the spread of coronavirus disease-2019 (“COVID-19”) in Hong Kong (as of 8, April 2020), see https://www.legco.gov.hk/yr19-20/english/panels/hs/papers/hs20200408cb2-794-1-e.pdf. A chronology of major measures is also shown in Fig. 1 in this paper.

3 The strict border restriction policy for travelers from the mainland was first extended for one month (from May 7 to June 7), and later extended a further three times (from June 7 to July 7, to August 7, then to September 7). As this paper is being finalized, it is still in effect and there may be continued extensions.

4 For details about the operational principles of Empirical Bayesian kriging method in Geostatistical Analyst in ArcGIS, see https://desktop.arcgis.com/en/arcmap/latest/extensions/geostatistical-analyst/what-is-empirical-bayesian-kriging-.htm

5 Hong Kong had 26 cumulative infections on February 8 and 107 cases on March 6. During this 28-day period, the daily growth rate in HK is denoted by ${x}_{hk}$ and can be computed from ${26\left(1+{x}_{hk}\right)}^{28-1}=107$. Similarly, the daily growth rate in the synthetic control unit is denoted as ${x}_{synthetic}$ and can be computed from ${25.254\left(1+{x}_{synthetic}\right)}^{28-1}=29.996$ for Panel A and ${24.566\left(1+{x}_{synthetic}\right)}^{28-1}=36.401$ for Panel B. Hence, the border restriction policy is associated with a difference of ${{x}_{hk}-x}_{synthetic}$ in the infection rate.

6 In Hong Kong, efforts on body temperature checking were concentrated at border checkpoints as well as the airport at that time. There was a lack of body temperature monitoring and other social distancing measures carried out in public space and workplaces at the time. The official direction for conducting body temperature screening at catering premises was issued on March 27 by the Secretary for Food and Health (SFH). Please refer to https://www.info.gov.hk/gia/general/202003/27/P2020032700878.htm?fontSize=1.
