# Supplementary material for: Evaluating the effectiveness of Hong Kong’s border restriction policy in reducing COVID-19 infections
Source: BMC Public Health. 2022 Apr 21;22:803. doi: 10.1186/s12889-022-13234-5 (PMC9023047; doi:10.1186/s12889-022-13234-5)
Supplement: Supplementary file 1 — Additional file 1: Descriptive statistics [file 12889_2022_13234_MOESM1_ESM.docx]

**Appendix**

**Descriptive Statistics**

| Variable | Obs | Mean | Std. Dev. | Min | Max |
| --- | --- | --- | --- | --- | --- |
| Number of daily cumulative infections | 37376 | 0.813 | 7.505 | 0 | 424 |
| Number of daily new infections | 37376 | 93.688 | 313.394 | 0 | 3518 |
| Past-14-day moving average of cumulative infections | 37376 | 87.584 | 302.287 | 0 | 3518 |
| Past-14-day moving average of cumulative infections per 10,000 people | 37376 | .893 | 5.167 | 0 | 83.057 |
| Population inflow (as of March 06) | 12306 | 33613 | 52275 | 472 | 591431 |
| Population outflow (as of March 06) | 12306 | 33036 | 33700 | 373 | 701168 |
| GDP (million, Chinese Yuan equivalent) | 41606 | 199396.35 | 418917.88 | 3586.07 | 3267987 |
| GDP per capita (Chinese Yuan equivalent) | 41606 | 76494.704 | 48173.079 | 19031 | 545738.95 |
| Population density (number of people per sq.km) | 41606 | 871.596 | 1442.304 | 4.884 | 21668.832 |
| Number of hospital beds per 10,000 people | 41606 | 79.269 | 33.227 | 3.75 | 203.053 |
| Number of medics per 10,000 people | 41606 | 39.665 | 16.341 | 2.417 | 99.123 |
| Daily average temperature  (℃) | 37376 | 12.257 | 9.082 | -28.135 | 31.571 |
| Relative humidity (%) | 37376 | 66.956 | 18.591 | 7.895 | 100 |
| Wind Speed (m/s) | 37376 | 2.416 | .902 | .653 | 12.083 |
| Air Quality Index (AQI) | 36936 | 59.422 | 32.938 | 9.083 | 500 |
|  | | | | | |
